# A taxonomy for the financing of health for all

**DOI:** 10.2471/BLT.24.291362

**Published:** 2024-03-04

**Authors:** Vanessa Huang, Maksym Obrizan, Juan Jardon-Pina

**Affiliations:** a Strand 50, 60 Bonham Strand, Sheung Wan, Hong Kong Special Administrative Region, China.; b Kyiv School of Economics, Kyiv, Ukraine.; c Saïd Business School, University of Oxford, Oxford, England.

The coronavirus disease 2019 (COVID-19) pandemic, increasingly frequent and severe natural disasters, and geopolitical events have highlighted the need for health systems that are resilient and can be rebuilt efficiently under adverse circumstances. To better prepare the world for the next health challenge, a whole-of-society approach that includes both government stewardship and private sector participation is key. Public and private financing of and investments in the health system should be actively and consistently leveraged to achieve the goals of health for all and global health equity. Drawing lessons from the climate movement’s progress in mobilizing governments and civil society, here we propose developing a health taxonomy with impactful and measurable health metrics, similar to the European Union (EU) taxonomy (Box 1) that was developed to support the climate movement. We explain why a health taxonomy is essential to mobilize health financing and investments from the private sector in addition to public funding.

Box 1 **The EU taxonomy**

The EU taxonomy regulation was published in the Official Journal of the European Union on 22 June 2020 and entered into force on 12 July 2020. Article 9 of the regulation sets out six climate and environmental objectives:

- Climate change mitigation
- Climate change adaptation
- Sustainable use and protection of water and marine resources
- Transition to a circular economy
- Pollution prevention and control
- Protection and restoration of biodiversity and ecosystems

The regulation also sets out four overarching conditions that an economic activity must meet to qualify as environmentally sustainable:

- Making a substantial contribution to at least one environmental objective
- Doing no significant harm to any of the other five environmental objectives
- Complying with minimum safeguards
- Complying with the technical screening criteria set out in the taxonomy delegated acts

To ensure that an economic activity substantially contributes to one of the six objectives while not doing significant harm to any of the other five objectives, the EU sets performance criteria called technical screening criteria in delegated acts. So far, criteria have been set for economic activities that can make a substantial contribution to climate change mitigation and climate change adaptation.

An example of an economic activity as defined by the EU taxonomy is given in Table 1.

EU: European Union

## Private sector participation 

Most private sector health-care companies develop pharmaceuticals, medical devices, health insurance and health technology products, which are the tools medical professionals use. Many of the world’s pharmaceutical products and medical devices are developed by companies in the United States of America and sold globally. Availability of the right tools that address local needs is a critical component to a resilient health system and to improving health equity. For example, the first generation of messenger ribonucleic acid (mRNA) COVID-19 vaccines required ultra-cold chain logistics, creating challenges for many low-income countries and remote delivery. The companies were able to subsequently develop a room-temperature-stable version, eliminating the problem. When they recognize public health needs, companies can actively contribute to health equity by developing better tools.

Financial market investments in the health-care sector are substantial. In the United States alone, these investments amounted to over 9.2 trillion United States dollars (US$) as of February 2024, including US$ 7.6 trillion in value in publicly listed health-care companies^1^ and US$ 1.6 trillion in health-care corporate debt.^2^ However, given that most financial investors are not public health experts, their health-themed investment decisions tend to be based on a company’s business performance and are often limited to traditional health-care companies. Expanded knowledge can broaden investors’ scope, guiding the pool of funding to support a wider range of companies that have a positive impact on public health.

The priorities and nomenclature private sector health-care investors and companies use often differ from those of policy-makers. The overarching goal for public health officials is to maximize the well-being of populations in a cost-efficient manner. On the other hand, private sector health-care investors and companies often pursue product innovation and scientific soundness, under the assumption that doctors will use regulatory-approved medical products, leading to financial returns. Health advocates should make efforts to facilitate the intersection of the private and public sectors to mobilize maximum financial resources, and to catalyse product innovations that can enhance health system resilience and global health equity.

## Case study: EU taxonomy

The climate movement is a powerful case study for health advocates on how to effectively mobilize and motivate both public funding and civil society investments towards common goals. Lessons from the climate movement show that well-studied metrics that are consistently measurable in a straightforward manner and comparable across countries, such as carbon emission reduction and water usage, are crucial in highlighting a company’s broader societal and population impact. With adequate information, stakeholders such as companies, investors, policy-makers and consumers can recognize and support climate-positive projects, and private sector funding can be mobilized.

To guide stakeholders to make sustainable investment decisions, the European Commission developed the EU taxonomy, a classification system that helps companies and investors identify environmentally sustainable economic activities. As of February 2024, the EU taxonomy covers economic activities in 17 sectors. Table 1 shows an example of the EU taxonomy as related to residential care activities.^3^

**Table 1:** Example in the human health and social work activities sector, from the EU taxonomy navigator

Activity	Substantial contribution criteria: climate adaptation	Do no significant harm on				
		Climate mitigation	Water	Circular economy	Pollution prevention	Bio-diversity
Residential care activities	The economic activity has implemented physical and non-physical solutions (adaptation solutions) that substantially reduce the most important physical climate risks that are material to that activity	N/A	N/A	N/A	A waste management plan is in place and ensures: (i) the safe and environmentally-sound handling of hazardous waste (in particular toxic or infectious waste) and pharmaceuticals; and (ii) maximal reuse or recycling of non-hazardous waste, including through contractual agreements with waste management partners	N/A

N/A: not applicable.

Note: Adapted from EU taxonomy navigator.^3^

The EU taxonomy allows for an easy and accurate incorporation of climate objectives in various sustainable investment frameworks. These climate-focused investment frameworks raised awareness about the climate impacts of different economic activities and corporate responsibilities, and mobilized significant private sector investments.

## Call for a health taxonomy

Similar global efforts are needed to develop a health taxonomy. A health taxonomy, akin to the EU taxonomy, could classify economic activities based on their impact against a set of common public health priorities. A health taxonomy can serve as a common language to educate stakeholders on health-positive and health-negative economic activities, and be a guide to steer public funding, private investments and policy decisions towards health-positive initiatives.

The health-related metrics currently used in various investment frameworks are often limited to company specifics, such as employee insurance coverage and injury rates. These metrics do not fully reflect the public health impacts of the company’s economic activities. Under a health taxonomy, a food company reducing chemicals used in its products can be classified as engaging in health-positive economic activities, similar to companies reducing carbon emissions. With a clearly defined health contribution of each industry’s economic activities, private sector health-targeted investments can expand beyond the traditional health-care sector and can include all companies that affect public health, such as companies that provide clean water facilities for remote areas. 

Specific health metrics should reflect a comprehensive view of individual and societal health status and the progress towards common health targets. Potential metrics include environmental health indicators such as pollution and water quality indices; percentage increase in access to primary health-care services; trackers of health outcomes across different populations; percentage reduction in medical errors; and public awareness levels of mental health. Similar to climate metrics, the health metrics should be comparable across countries and measurable in a straightforward manner.

## Operationalizing a health taxonomy 

A well-developed health taxonomy will enable sustainable investment frameworks such as environment, social and governance investing initiatives to incorporate public health objectives easily and accurately, since health naturally falls under the social category.^4^ In recent years, environment, social and governance-themed investing has been of notable interest to financial investors. The Principles for Responsible Investment initiative, which is often used as a gauge for environment, social and governance-committed capital, has reached over 5300 signatories representing over US$ 120 trillion in assets under management (that is, total fund amount) as of September 2023.^5^ Moreover, health is a key element of the United Nations sustainable development goals (SDGs). The health taxonomy can also be used to show alignment between economic activities and the SDGs to allow more effective allocation of resources and better tracking of progress.

Consistently integrating health metrics into sustainable investment frameworks will increase awareness and mobilize private sector funding and investments into health-positive companies and industries. As demonstrated by the climate movement, access to additional funding can provide incentives to companies to incorporate public health into their company strategies and product innovation pipelines. Increased investments from companies and investors can in turn stimulate advancement in the respective sectors, potentially leading to economic growth and job creation, as seen in the growth of alternative energy ecosystems globally. More targeted and effective use of health resources, in the long run, could lead to improved general health, reduced incidence of certain diseases, strengthened health system resilience and improved health equity. 

## World Health Organization’s role 

Research from the World Health Organization (WHO) shows that social factors can be more important than health care or lifestyle choices in influencing health, with some studies suggesting that social factors account for 30%–55% of health outcomes.^6^ A global panel of health experts is needed to develop a set of globally relevant health targets that considers macro and national level nuances, and recommend a set of common health metrics that are impactful and measurable. Different approaches can be taken to initiate work on a health taxonomy, ranging from an academic-led effort to an initiative driven by the financial industry. However, WHO’s domain knowledge, global representation, convening power and existing work on the commercial determinants of health,^7^ makes it the ideal organization to spearhead the development of the health taxonomy.

WHO can propose a resolution that backs the creation of a health taxonomy, which Member States can support and work towards implementing in their own national contexts. After identifying the health targets and metrics, WHO can drive a process similar to that of the drafting of the EU taxonomy, with governments, health experts, global entities and the private sector, to develop a classification of economic activities that contribute positively or negatively to these health targets. A health taxonomy and metrics developed by a WHO-led global consortium will ensure credibility, promote adoption, ensure transparency and raise awareness of the public health impacts of different economic activities. Doing so can also encourage global collaboration and standardization in measuring and reporting on health impacts, preventing potential health-washing (that is, when a person falsely presents an activity as health-positive), and may result in prioritization of funding for better health initiatives such as preventative care. Consumers will be better informed and can make appropriate choices that can have long-term impacts on their health.

## Conclusion

A well-developed health taxonomy is needed to make more progress on public health and global health equity goals. A health taxonomy can align economic activities with public health priorities, incentivizing both public and private investments towards impactful health initiatives. For a health taxonomy to be globally significant, it is crucial to identify and prioritize economic activities that strengthen health system resilience and advance health equity within and between countries. Coordinated efforts are essential for the development, adoption and implementation of the health taxonomy. A health taxonomy is more than a measurement tool; it can become a driving force for a whole-of-society mobilization to achieve health for all.
